# Lipoprotein modifications by gingipains of *Porphyromonas gingivalis*

**DOI:** 10.1111/jre.12527

**Published:** 2018-01-17

**Authors:** J. Lönn, S. Ljunggren, K. Klarström‐Engström, I. Demirel, T. Bengtsson, H. Karlsson

**Affiliations:** ^1^ Department of Oral Biology Institute of Odontology Malmö University Malmö Sweden; ^2^ PEAS Institute AB Linköping Sweden; ^3^ Department of Clinical and Experimental Medicine Occupational and Environmental Medicine Center Linköping University Linköping Sweden; ^4^ Department of Medical Sciences Örebro University Örebro Sweden

**Keywords:** gingipains, lipoproteins, MALDI‐TOF mass spectrometry, *Porphyromonas gingivalis*, two‐dimensional gel electrophoresis

## Abstract

**Background and Objective:**

Several studies have shown an association between periodontitis and cardiovascular disease (CVD). Atherosclerosis is the major cause of CVD, and a key event in the development of atherosclerosis is accumulation of lipoproteins within the arterial wall. Bacteria are the primary etiologic agents in periodontitis and *Porphyromonas gingivalis* is the major pathogen in the disease. Several studies support a role of modified low‐density lipoprotein (LDL) in atherogenesis; however, the pathogenic stimuli that induce the changes and the mechanisms by which this occur are unknown. This study aims to identify alterations in plasma lipoproteins induced by the periodontopathic species of bacterium, *P. gingivalis*, in vitro.

**Material and Methods:**

Plasma lipoproteins were isolated from whole blood treated with wild‐type and gingipain‐mutant (lacking either the Rgp‐ or Kgp gingipains) *P. gingivalis* by density/gradient‐ultracentrifugation and were studied using 2‐dimensional gel electrophoresis followed by matrix‐assisted laser desorption/ionization mass spectrometry. *Porphyromonas gingivalis*‐induced lipid peroxidation and antioxidant levels were measured by thiobarbituric acid‐reactive substances and antioxidant assay kits, respectively, and lumiaggregometry was used for measurement of reactive oxygen species (ROS) and aggregation.

**Results:**

*Porphyromonas gingivalis* exerted substantial proteolytic effects on the lipoproteins. The Rgp gingipains were responsible for producing 2 apoE fragments, as well as 2 apoB‐100 fragments, in LDL, and the Kgp gingipain produced an unidentified fragment in high‐density lipoproteins. *Porphyromonas gingivalis* and its different gingipain variants induced ROS and consumed antioxidants. Both the Rgp and Kgp gingipains were involved in inducing lipid peroxidation.

**Conclusion:**

*Porphyromonas gingivalis* has the potential to change the expression of lipoproteins in blood, which may represent a crucial link between periodontitis and CVD.

## 1 INTRODUCTION

Periodontitis is one of the most common chronic inflammatory diseases in the world and there is emerging interest in the link between oral health and cardiovascular disease (CVD). Indeed, CVD and mortality are more prevalent among patients with periodontitis than without periodontitis, and periodontal infection is strongly associated with CVD risk.^1^, ^2^, ^3^ Atherosclerosis is the major cause of CVD, and a key event in this inflammatory process is accumulation of lipoproteins within the arterial wall.^4^ Modified low‐density lipoprotein (LDL) exerts various effects supporting atherogenesis, including increased expression of adhesion molecules, leukocyte adhesion and chemotaxis, injury to endothelial cells, enhanced foam cell formation, increased smooth muscle cell and fibroblast proliferation and release of inflammatory mediators.^5^, ^6^

A dysbiotic bacterial community is the primary etiologic agent in periodontitis, and *Porphyromonas gingivalis* is considered to be the key pathogen in periodontal disease, mainly because of its potent virulence factors, the gingipains, that are proteolytic enzymes capable of degrading host proteins.^7^ Bale et al^8^ propose that periodontal disease induced by high‐risk pathogens, including *P. gingivalis*, may be considered as a contributory cause of arterial disease, although the evidence is not clear.

In periodontitis, the host–pathogen interaction initiates a destructive inflammatory response, eventually exposing the bacteria to the bloodstream as a result of ulceration of the gingival epithelium. Oxidative stress, such as over‐production of reactive oxygen species (ROS), and expression of proteolytic enzymes (bacteria‐ and host‐derived) play crucial roles in the chronic inflammatory reactions of periodontitis and atherosclerosis.^9^, ^10^ *Porphyromonas gingivalis* expresses a broad range of virulence factors, including lipopolysaccharides (LPS), fimbriae, adhesins, hemagglutinins and an array of proteolytic enzymes.^11^ Among these factors the gingipains are essential both for bacterial survival and for the pathological outcome. Two types of cysteine proteases are responsible for the so‐called trypsin‐like activity of *P. gingivalis* – lysine‐specific (Kgp) and arginine‐specific (Rgp) gingipains, as determined by the specificity of their cleaving sites. These proteases have disruptive effects on inflammatory cells, modulate blood coagulation, enhance vascular permeability and activate the complement system.^12^

The mechanisms involved in the development of atherosclerosis, including the role of periodontitis and periodontal pathogens, are far from clearly understood. Although several studies support a crucial role of modified LDL in atherogenesis, the pathogenic stimuli that induce the changes and the mechanisms by which this occurs in vivo are still unknown. Data have also provided evidence of a direct relationship between periodontal microbes and subclinical atherosclerosis. *Porphyromonas gingivalis* (both DNA and live bacteria) has been found within atherosclerotic plaques and the innate and adaptive immune responses against the bacteria have been proposed to contribute to the development of atherosclerosis.^13^, ^14^, ^15^ Furthermore, experimental animal models demonstrate that infection with periodontal pathogens accelerates atherogenesis.^16^ However, the mechanisms by which infectious agents contribute to the development of atherosclerosis should be further evaluated as the host–pathogen interaction is not well studied. The infection can either play a role in initiating the disease or aggravate the already established inflammation of an atherosclerotic plaque.^10^, ^17^

Modified LDL has been suggested to be involved in the initial phase of atherosclerosis, as well as during its progression. Changes in endothelial permeability and composition of the extracellular matrix promote the entry of cholesterol‐containing LDL particles into the artery wall.^18^ Monocytes have a crucial role in inducing and maintaining the inflammatory process in the atherosclerotic plaque by recruiting other immune cells, internalizing modified LDL and forming foam cells. The retention of modified LDL in sub‐endothelial extracellular matrix and its internalization by monocytes/macrophages is a hallmark of atherosclerosis.^19^ High‐density lipoprotein (HDL) has anti‐atherogenic properties, primarily as a result of reverse cholesterol transport and by protecting lipoproteins from oxidation.^20^ Paraoxonase 1 (PON1) is an anti‐oxidative enzyme mainly found in HDL. Alterations in the level of PON1 in the circulation have been reported during oxidative stress and PON1 activity is involved in the defence of free radical production.^21^ When pathogenic or environmental stress elevates ROS production to an extent where the concentrations exceed the protective levels of antioxidants, this may result in the damage of lipids, proteins and DNA. LDL and other lipids (eg, in the cell membrane) can be oxidized by ROS or via enzymatic reactions^22^ and give rise to highly reactive by‐products, such as malondialdehyde (MDA). This process is termed lipid peroxidation and is, when in excess or unregulated, suggested to be an underlying mechanism of atherosclerosis and cancer.

The precise mechanisms responsible for the modifications of lipoproteins are not fully elucidated, therefore are qualitative alterations of LDL, as well as of HDL, important to investigate. We have previously shown that incubation of human whole blood with *P. gingivalis* causes formation of leukocyte/platelet aggregates and ROS production; furthermore, the bacteria induced alterations of LDL.^23^, ^24^ In the current study, we used different strains of *P. gingivalis*, lacking either the Rgp gingipains or the Kgp gingipains. We hypothesize that *P. gingivalis* triggers the formation of modified LDL, and possibly HDL, in whole blood through mechanisms involving gingipains. The aim of this study was to identify and characterize alterations of lipoproteins induced by *P. gingivalis* and its proteolytic enzymes.

## 2 MATERIAL AND METHODS

### 2.1 *Porphyromonas gingivalis*

*Porphyromonas gingivalis* wild‐type strains ATCC 33277 (American Type Culture Collection, Manassas, VA, USA) and W50, and the W50‐derived Rgp and Kgp protease mutant strains (E8 and K1A, respectively; a kind gift from Dr M. Curtis, Barts and The London, Queen Mary's School of Medicine and Dentistry, UK), were grown in anaerobic conditions (80% N_2_, 10% CO_2_ and 10% H_2_) at 37°C in an anaerobic chamber (Concept 400 Anaerobic Workstation; Ruskinn Technology Ltd., Leeds, UK). E8 is a double mutant which lacks RgpA and RgpB, whereas K1A lacks Kgp. The bacteria were cultured for 3 days in fastidious anaerobe broth (29.7 g/L, pH 7.2; Lab M Limited, Lancashire, UK) before being washed twice by centrifugation for 10 minutes at 10 000* g* at room temperature and resuspended in physiological (0.9%) NaCl. An optical density (OD) of 2.0 at 600 nm (BioPhotometer plus; Eppendorf AG, Hamburg, Germany) of the bacteria in suspension correlated to a colony‐forming unit (CFU) value of ~10^9^ after culture for 5‐7 days on fastidious anaerobe agar plates supplemented with defibrinated horse blood (5%).

### 2.2 *P. gingivalis* stimulation of whole blood and isolation of LDL/Very LDL and HDL

Peripheral venous blood was drawn from 6 fasting healthy (according to the regular dental check‐ups) donors at Örebro University Hospital, Sweden, and collected into heparin‐containing (20 U/mL) Vacutainer tubes. Blood samples were collected according to the Swedish National Board of Health and Welfare guidelines and the Ethical Guidelines of the Helsinki Declaration. The blood was preincubated for 10 minutes at 37°C under shaking in a water bath and thereafter unstimulated or stimulated with *P. gingivalis* (5 × 10^7^ CFU/mL of blood) for an additional 30 minutes. The plasma was collected after centrifugation (2200 *g* for 10 minutes at room temperature) of the blood, immediately frozen at −20°C, then removed and stored at −80°C until required for isolation of LDL and HDL.

The LDL fraction also contains very‐low‐density lipoprotein (VLDL). LDL/VLDL and HDL were prepared from the plasma by ultracentrifugation procedures, as previously described.^25^, ^26^ EDTA (1 mg/mL) and sucrose (0.5%) were added to plasma to prevent oxidation and aggregation. Five millilitres of plasma was adjusted to a density of 1.24 g/mL with solid potassium bromide (KBr) and placed in the bottom of a centrifuge tube (Beckman Coulter; Quick‐Seal Polyallomer, 16 × 76 mm, 13.5 mL; Beckman Instruments, Inc., Palo Alto, CA, USA), and KBr/phosphate buffer solution (1.063 g/mL) was carefully layered above the plasma without mixing of the phases. Ultracentrifugation was performed at 290 000 *g* for 4 hours at 15°C. The separate bands with LDL/VLDL at the top and HDL in the middle were collected with a syringe and transferred to new centrifuge tubes. KBr solution (1.24 g/mL) was added and the preparations were then centrifuged for 2 hours at 15°C. LDL/VLDL and HDL, almost depleted of albumin and other plasma contaminants, were then extracted from the top of the tube and desalted in PD 10 columns (GE Healthcare, Little Chalfont, UK) and stored in 3.5 mL of desalting buffer (CH_5_NO_3_, 12 mmol/L, pH 7.1) at −70°C until lyophilized. The protein concentration of the preparations was determined with a 2D Quant‐kit protein assay (GE Healthcare).

### 2.3 Two‐dimensional gel electrophoresis

Proteins of the lipoprotein particles were separated as described previously.^25^ In the first dimension, 300 μg of protein was isoelectrically focused in immobilized pH gradient strips (pH 3‐10) at 48 000 volt‐hours (Vhr) (maximum: 8000 V) using the isoelectric focusing system IPGphor (Pharmacia Biotech, Uppsala, Sweden).

In the second dimension, SDS‐PAGE was performed by transferring the proteins to a homogenous (T [gel concentration] = 14%, C [crosslinking] = 1%) home cast gel on gel bond, which was run at 60‐800 V, 10°C, 30 mA overnight in the electrophoresis system Multiphor (Pharmacia Biotech). Separated proteins were then fixed and visualized by silver staining,^27^ with a detection limit of about 5 ng/spot.^28^ The protein spots on gel images were quantified as OD using PDQuest 2‐D gel analysis software version 7.1 (Bio‐Rad Laboratories AB, Solna, Sweden) and the expression is presented in PPM‐values (parts per million) of the total gel OD.

### 2.4 Tryptic digestion, mass spectrometry and database search

Protein spots were picked out from the gels and destained with 30 mmol/L of potassium ferricyanide/100 mmol/L of sodium thiosulfate and thereafter washed in 200 mmol/L of ammonium bicarbonate, dehydrated with 100% acetonitrile and dried by vacuum centrifugation (SpeedVac; Savant, Farmingdale, NY, USA). The dried gel pieces were incubated overnight at 37°C with trypsin (10 μg/mL; Promega, Madison, WI, USA) diluted in 25 μmol/L of ammonium bicarbonate. The supernatant was transferred to a new vial and dried by vacuum centrifugation, and the peptides were further extracted from the gel piece by incubation in 50% acetonitrile/5% trifluoroacetic acid for approximately 3 hours at room temperature with continuous mixing. The supernatants obtained from the 2 steps were pooled and again dried by vacuum centrifugation, and the peptides were dissolved in 0.1% trifluoroacetic acid. Then, the peptides were mixed 1:1 with matrix solution (50 mg/mL of 2,5‐dihydroxybenzoic acid in 70% acetonitrile/0.3% trifluoroacetic acid) and then spotted onto a stainless‐steel target plate. Analyses of peptide masses were performed using MALDI‐TOF mass spectrometry (Voyager DE PRO; Applied Biosystems, Foster City, CA, USA).

NCBI and SWISS‐PROT were used with MS‐Fit (ProteinProspector, University of San Francisco) as search engines. Restrictions were human species, mass tolerance <50 p.p.m., maximum one missed cleavage by trypsin, methionine oxidation and cysteine modification by carbamidomethylation.

### 2.5 Methionine oxidation of apolipoprotein A‐1 in HDL

The effect of *P. gingivalis* on methionine oxidation was analysed by calculating the ratio of the peptide intensity of the oxidized and nonoxidized methionines at positions 136 (*m*/*z* 1047 and 1031) and 172 (*m*/*z* 1299 and 1283) in the mass spectrum data of apolipoprotein A‐1 (apoA‐1) of HDL (the positions prone to oxidation have been described previously in ref. 29).

### 2.6 PON1 arylesterase activity

The activity of PON1 arylesterase activity was measured kinetically by first diluting the plasma at a ratio of 1:80 in a salt buffer containing 20 mmol/L of Tris‐HCl and 1.0 mmol/L CaCl_2_ in water, to activate the enzyme. Twenty microlitres of the diluted plasma was then mixed with 200 μL of phenyl acetate solution, containing 3.26 mmol/L of phenyl acetate in salt buffer. The phenol produced was measured at 270 nm, with 250 nm as background, using a spectrophotometer (SpectraMax 190 plate reader; Molecular Devices, Sunnyvale, CA, USA) and the activity was calculated using the extinction coefficient of phenol of 1310 M/cm and expressed in μmol/min/mL (U/mL).^29^

### 2.7 Reactive oxygen species production

Reactive oxygen species production and aggregation were measured using a lumiaggregometer model 560 (Chrono‐Log Corp., Havertown, PA, USA). Cell aggregation was registered as an increase in impedance (Ω) between 2 platinum electrodes, and ROS production was determined simultaneously through luminol‐amplified chemiluminescence, as previously described.^24^ Briefly, heparinized whole blood was diluted at a ratio of 1:1 in physiological sodium chloride (0.9% NaCl) and preincubated with luminol (0.2 mg/mL; Sigma Aldrich, St Louis, MO, USA) and horseradish peroxidase (8 U/mL; Sigma Aldrich) for 15 minutes at 37°C in plastic cuvettes with siliconized stirring bars rotating at 800 rpm. *Porphyromonas gingivalis* ATCC33277 (1 × 10^7^ CFU/mL of blood) was preincubated in the presence or absence of leupeptin (which inhibits serine and cysteine proteases, and thus acts on the Rgp gingipains; 0.1 mmol/L; Roche Diagnostics Co., Indianapolis, IN, USA) before addition to the blood and incubated for 25 minutes. ROS production induced by W50, K1A, E8 or LPS (1 μg/mL) was also measured.

### 2.8 Antioxidant assay

Whole blood was challenged without or with bacteria (1 × 10^6^ CFU/mL of blood) for 3, 20 and 60 minutes. LPS (1 μg/mL) was used as a positive control. Samples were centrifuged for 15 minutes at 1000 *g*, room temperature, to obtain plasma. Supernatants were partitioned and immediately frozen and stored at −80°C until required for analysis. Samples were analysed for total antioxidant capacity using an antioxidant assay kit according to the manufacturer's instructions (Sigma Aldrich). Briefly, metmyoglobin and H_2_O_2_ form a ferryl myoglobin radical that oxidizes ABTS (2,2′‐azino‐bis(3‐ethylbenzthiazoline‐6‐sulfonic acid)) to a green radical cation. Any antioxidants present in the sample will suppress the formation of the green radical in a concentration‐dependent manner. Absorbance was read at 450 nm using an ELISA plate reader.

### 2.9 Thiobarbituric acid reactive substances assay for lipid peroxidation analysis

Samples from the same preparation as for the antioxidant assay were used to analyse lipid peroxidation with a thiobarbituric acid reactive substances (TBARS) assay by measuring plasma concentrations of MDA, a by‐product formed in the lipid peroxidation process. Briefly, samples were mixed with butylated hydroxytoulene (0.1%) and NaOH (2.5 mmol/L) and incubated in a water bath at 60°C for 30 minutes. Trichloroacetic acid (7.2%) supplemented with 1% potassium iodide was added to the samples, which were then centrifuged at 9000 *g* for 10 minutes. Supernatants were mixed with thiobarbituric acid and incubated at 90°C for 40 minutes. Thiobarbituric acid forms a red, fluorescent adduct together with 2 molecules of MDA, and is thus quantifiable when compared with MDA standards prepared from 1,1,3,3‐tetraethoxypropane (Sigma Aldrich). A FLUOstar plate reader was used to detect emission at 590 nm at an excitation of 544 nm (BMG Labtechnologies, Offenburg, Germany).

### 2.10 Statistical analyses

The quantification data were not normally distributed and therefore analysed using either Friedman's two‐way ANOVA and the Wilcoxon signed‐rank test for HDL, where data were paired, or the Kruskal‐Wallis test followed by the Mann‐Whitney *U*‐test for LDL, where unpaired data were included, using SPSS (SPSS Inc., New York, NY, USA) as well as GraphPad Prism (GraphPad Software, La Jolla, CA, USA). Values are expressed as mean ± standard error of the mean, and *P* ≤ .05 was considered as statistically significant. Significance is denoted as: *P < .05, **P < .01 and ***P < .001.

## 3 RESULTS

### 3.1 *P. gingivalis* modifies the expression of LDL/VLDL and HDL

The effects of *P. gingivalis* on the expression of LDL/VLDL and HDL protein were studied using 2‐dimensional gel electrophoresis (2DE). LDL/VLDL and HDL were isolated from *P. gingivalis*‐stimulated (30 minutes, 5 × 10^7^ CFU/mL, 37°C, shaking) or unstimulated (30 minutes, 37°C, shaking) whole blood.

### 3.2 LDL/VLDL

**Figure 1:**
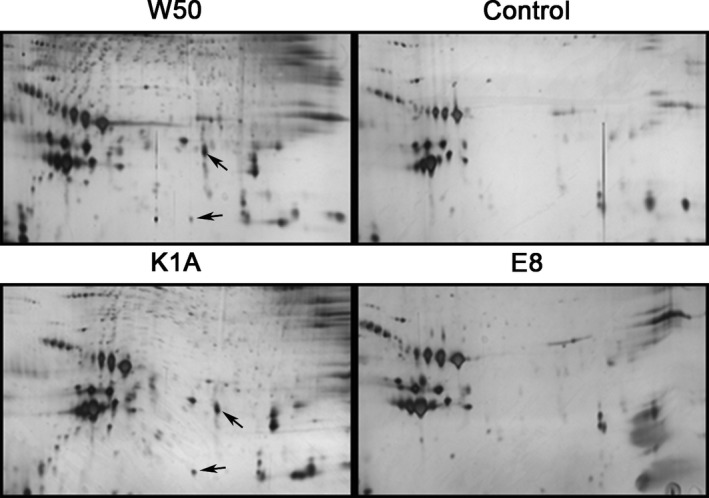
*Porphyromonas gingivalis* produced additional protein spots in the silver‐stained 2‐dimensional gel electrophoresis (2DE) pattern of low‐density lipoprotein/very‐low‐density lipoprotein (LDL/VLDL). The protein pattern from whole blood exposed to the W50 wild‐type strain of *P. gingivalis*, the gingipain mutants E8 (lacking RgpA and RgpB) and K1A (lacking Kgp) (5 × 10^7^ colony‐forming units [CFU]/mL at 37°C, 30 minutes) and the unstimulated control from the same subject are shown. Arrows indicate two non‐identified fragments

By comparing the results with previous findings,^23^, ^25^ most of the proteins detected in LDL/VLDL were identified as apolipoproteins, including apoA‐1, apoM, apoE, apoJ, apoA‐4 and serum amyloid A (SAA)_4_. ApoB‐100, the most abundant lipoprotein in LDL, was not detected in its full‐length form in the 2DE pattern from untreated samples because of its large size (500 kDa) and hydrophobic characteristics. *Porphyromonas gingivalis* wild‐type strains (ATCC and W50) produced numerous additional protein spots in the gels (Figure 1, W50 shown). The Kgp mutant strain, K1A, produced a protein expression pattern in the gel which was similar to that of the wild‐type strains, whereas the 2DE pattern of protein expression in the gels of the Rgp mutant strain, E8, corresponded more to the pattern of the unstimulated control (Figure 1).

**Figure 2:**
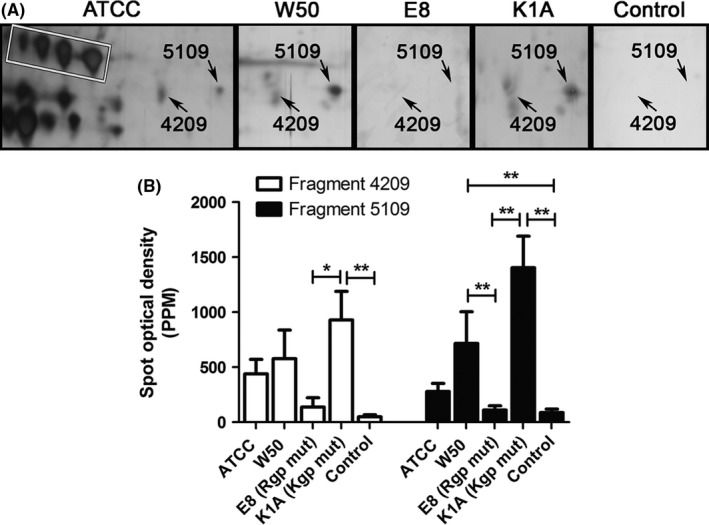
Apolipoprotein E (apoE) fragmentation in low‐density lipoprotein/very‐low‐density lipoprotein (LDL/VLDL) isolated from *Porphyromonas gingivalis*‐treated whole blood. Whole blood was incubated with *P. gingivalis* (5 × 10^7^ colony‐forming units [CFU]/mL, at 37°C, 30 minutes) wild‐type strains ATCC or W50, with the gingipain mutants E8 (lacking RgpA and RgpB) and K1A (lacking Kgp) or without bacteria (Control). LDL was isolated from plasma by density/gradient‐ultracentrifugation and proteins were separated with 2‐dimensional gel electrophoresis (2DE) and silver stained (A). Two apoE fragments (4209: p*I* 5.9, M*r* 32 kDa; and 5109: p*I* 6.1, M*r* 32 kDa) were identified by mass‐spectrometry analysis. The standard positions of apoE are shown in the white box. B, The mean ± standard error of the mean spot optical density in parts per million (PPM) of the total gel staining is presented from at least 4 independent experiments/subjects (n = 4‐6,) (*P < .1; **P < .01; ***P < .001). mut, mutant

**Figure 3:**
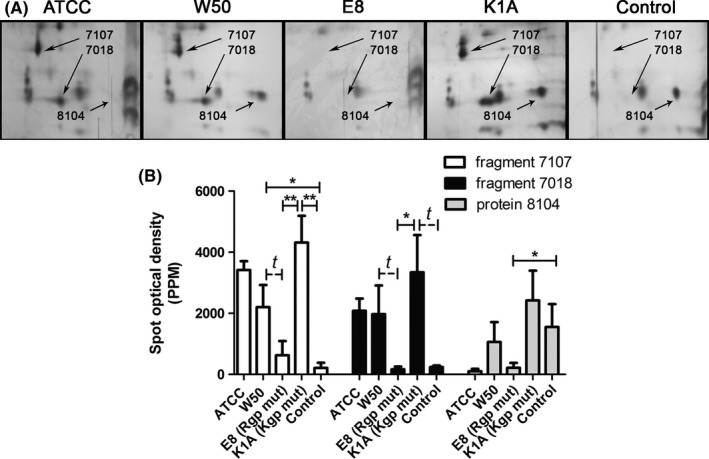
Apolipoprotein B‐100 (apoB‐100) fragmentation and expression of serum amyloid A (SAA)_4_ in low‐density lipoprotein/very‐low‐density lipoprotein (LDL/VLDL) isolated from *Porphyromonas gingivalis*‐treated whole blood. Whole blood was incubated with *P. gingivalis* (5 × 10^7^ colony‐forming units [CFU]/mL, at 37°C, 30 minutes) wild‐type strains ATCC or W50, with the gingipain mutants E8 (lacking RgpA and RgpB) and K1A (lacking Kgp) or without bacteria (Control). LDL/VLDL was isolated from plasma by density/gradient‐ultracentrifugation and proteins were separated with 2‐dimensional gel electrophoresis (2DE) and silver stained (A). Two apoB‐100 fragments (7107: p*I* 7.2, M*r* 20 kDa; and 7018: p*I* 7.9, M*r* 15 kDa) and one SAA_4_ isoform (8104: p*I* 9.17, M*r* 14.7 kDa) were identified by mass‐spectrometry analysis. B, The mean ± standard error of the mean spot optical density in parts per million (PPM) of total gel staining is presented from at least 4 independent experiments/subjects (n = 4‐6) (*P < .05, **P < .01, and ^*t*^ *P ≤ *.06). mut, mutant

Four specific fragments were identified by mass spectrometry in LDL/VLDL isolated from *P. gingivalis‐*treated whole blood (Table S1): 2 apoE C‐terminal fragments (spot numbers: 4209 and 5109, Figure 2A,B) with a molecular mass of 32 kDa each and p*I* 5.9 and 6.0, respectively, and 2 apoB‐100 N‐terminal fragments (spot numbers: 7107 and 7018, Figure 3A,B) with molecular mass values of 20 and 15 kDa, and p*I* 7.2 and 7.9, respectively. The molecular masses and amino acid sequences of the apoE fragments indicate that they originate from apoE‐3 (Table S2). No fragmentation was seen in LDL/VLDL from the blood stimulated with E8. However, E8 significantly reduced the expression of one serum amyloid A (SAA)_4_ isoform (spot number 8104, Figure 3A,B). The expression of one additional SAA_4_ isoform was reduced although no significant difference was found (*P *= .06).

An additional 2 protein spots/fragments (indicated by arrows in Figure 1) were induced by *P. gingivalis* in a similar pattern as the expression of the apoE and apoB fragments. The Kgp mutant K1A significantly increased the expression of these fragments compared with the control and E8. However, the mass‐spectrometry analysis was unable to identify the proteins because of the low amount of protein and small numbers of peptides present.

### 3.3 High‐density lipoprotein

**Figure 4:**
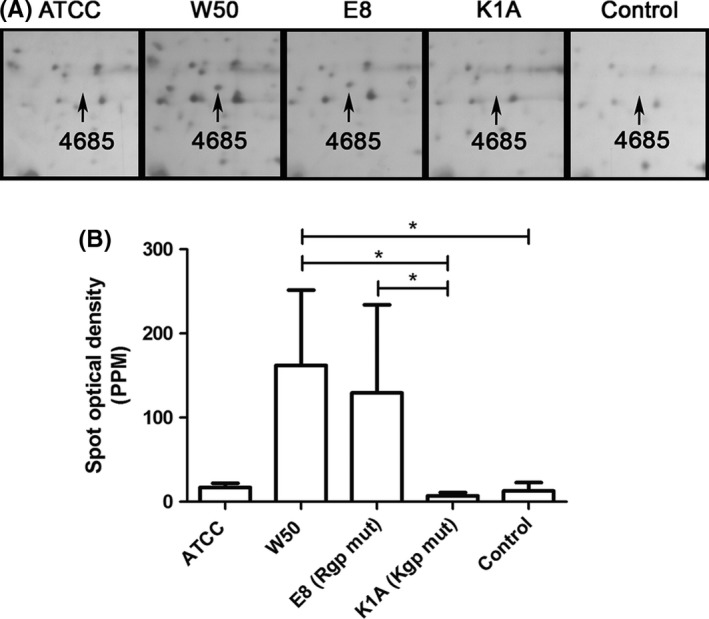
A lysine gingipain‐dependent fragment (4685) was found in high‐density lipoprotein (HDL) isolated from *Porphyromonas gingivalis*‐stimulated whole blood. Whole blood was incubated with *P. gingivalis* (5 × 10^7^ colony‐forming units [CFU]/mL, at 37°C, 30 minutes) wild‐type strains ATCC or W50, with the gingipain mutants E8 (lacking RgpA and RgpB) and K1A (lacking Kgp) or without bacteria (Control). Low‐density lipoprotein (LDL) was isolated from plasma by density/gradient‐ultracentrifugation and proteins were separated using 2‐dimensional gel electrophoresis (2DE) and visualized by silver staining (A). B, Mean ± standard error of the mean spot optical density in parts per million (PPM) of total gel optical density is presented (n = 3) (*P < .05). mut, mutant

In HDL, *P. gingivalis* W50 produced one fragment that was considered to be dependent on the Kgp gingipain as it was expressed in E8 (spot number 4685 in Figure 4, just above the 3 apoL isoforms and at the same level as the head of the arrow) but not in K1A‐treated blood. The spot could not be identified by mass‐spectrometry analysis because of the low amount of protein and/or small numbers of peptides present. E8 decreased the expression of several proteins in HDL, including apoA‐4 (*P *= .053), as identified by map‐matching; however, these results were not statistically significant.

### 3.4 Methionine oxidation

**Figure 5:**
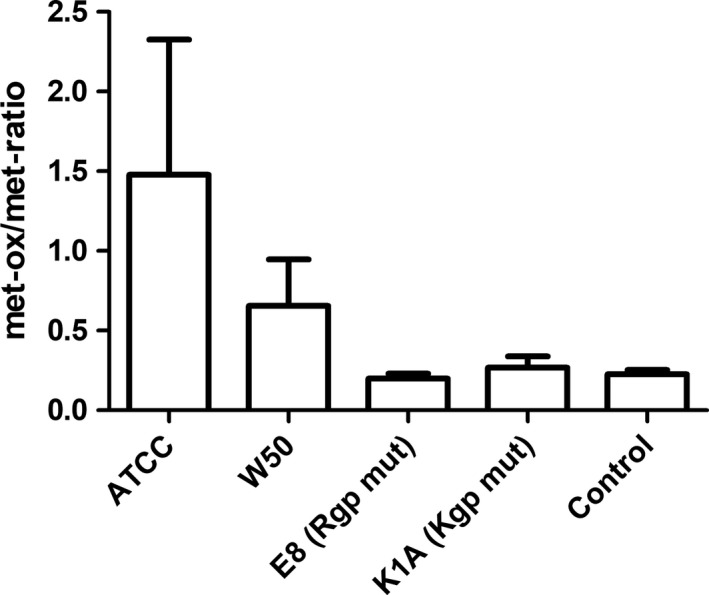
Methionine oxidation of apolipoprotein A‐1 (apoA‐1) in high‐density lipoprotein (HDL). HDL was isolated by density/gradient‐ultracentrifugation of plasma from whole blood treated with *Porphyromonas gingivalis* (5 × 10^7^ colony‐forming units [CFU]/mL, at 37°C, 30 minutes) wild‐type strains ATCC or W50, with the gingipain mutants E8 (lacking RgpA and RgpB) and K1A (lacking Kgp) or without bacteria (Control). The ratio of oxidized/nonoxidized peptide intensities of methionine at amino acid positions 137 and 172 in apoA‐1 were calculated from the mass‐spectrum data obtained after separating isolated HDL by 2‐dimensional gel electrophoresis (2DE) and silver staining. Data are expressed as mean ± standard error of the mean (n = 3). met‐ox, methionine oxidation; mut, mutant

There was no significant effect of *P. gingivalis* on methionine oxidation of apoA‐1 in HDL. However, a trend of increased methionine oxidation in HDL induced by the wild‐type strains (ATCC and W50), but not by E8 or K1A, was seen (Figure 5), indicating that both of the gingipains are necessary for methionine oxidation. When combining the intensities of the mass‐spectrometry data of the methionines at positions 136 and 172 on both HDL and LDL/VLDL, we found that the Rgp mutant E8 significantly decreased methionine oxidation.

### 3.5 *Porphyromonas gingivalis* does not affect PON1 arylesterase activity

*Porphyromonas gingivalis* did not show any effect on PON1 arylesterase activity in whole blood.

### 3.6 Leupeptin inhibits *P. gingivalis‐*induced ROS production and aggregation

**Figure 6:**
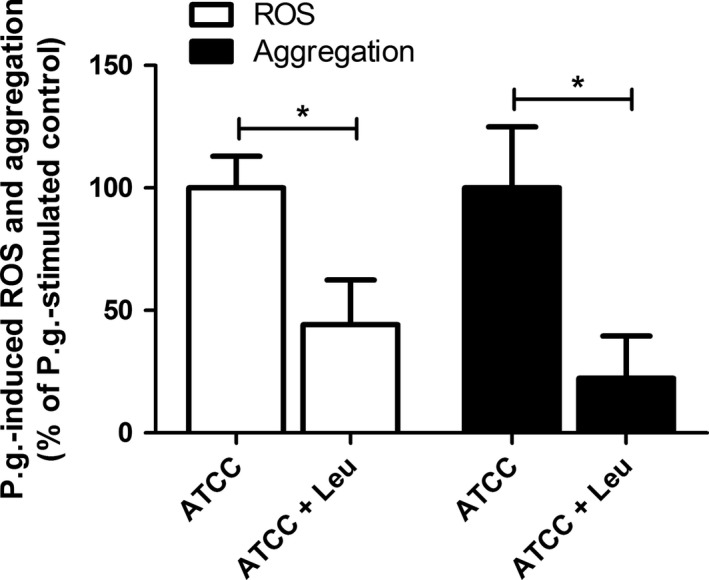
Leupeptin inhibits *Porphyromonas gingivalis*‐induced reactive oxygen species (ROS) production and aggregation in whole blood. Whole blood was incubated for 15 minutes at 37°C in the absence or presence of leupeptin (0.1 mmol/L), then stimulated with *P. gingivalis* (ATCC, 1 × 10^7^ colony‐forming units [CFU]/mL) for 25 minutes. ROS production was detected by luminol‐amplified chemiluminescence and aggregation was measured as changes in impedance, presented as percentage of *P. gingivalis*‐stimulated control. Data are expressed as mean ± standard error of the mean (n = 8) (*P < .05). Leu, leupeptin; P.g., *Porphyromonas gingivalis*

The *P. gingivalis* (ATCC33277)‐induced extensive ROS production and aggregation in whole blood were significantly inhibited by leupeptin (Figure 6). Stimulation with E8 or K1A, which express only one type of gingipain, also induced significant amounts of ROS; however, *P. gingivalis* LPS did not induce detectable amounts of ROS.

### 3.7 *Porphyromonas gingivalis* consumes antioxidants and induces lipid peroxidation

**Figure 7:**
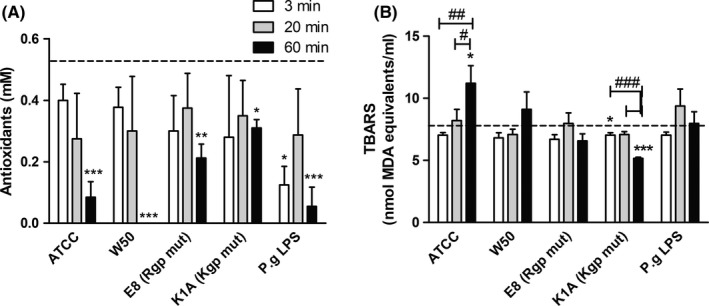
*Porphyromonas gingivalis* challenge of whole blood consumes antioxidants and causes lipid peroxidation. Whole blood was challenged with *P. gingivalis* (1 × 10^6^ colony‐forming units [CFU]/mL) for 3, 20 and 60 minutes, whereafter plasma was prepared and analysed for total antioxidant capacity (A) and lipid peroxidation (B). Dotted lines indicate basal level in blood. Data are expressed as mean ± standard error of the mean (n = 5). (*P < .05, **P < .01, and ***P < .001). LPS, lipopolysaccharide; mut, mutant; P.g., *Porphyromonas gingivalis*

Antioxidants protect cells from free radicals (eg, damage of the lipid integrity of the cell membrane). Total antioxidant capacity was measured in plasma collected from whole blood stimulated with the different *P. gingivalis* strains, or LPS. All stimuli caused a time‐dependent decrease in antioxidant levels with significant reductions observed after 60 minutes (Figure 7A).

To examine if *P. gingivalis* induces production of ROS and if antioxidant consumption affects lipid peroxidation, a TBARS assay was performed. *Porphyromonas gingivalis* wild‐type strains ATCC and W50 caused a time‐dependent increase in MDA concentration, reaching the highest levels after 60 minutes. These results correspond to the antioxidant data, indicating that antioxidants are used to prevent lipid peroxidation. On the contrary, stimulation of whole blood with the Kgp mutant, K1A, decreased the amounts of measurable MDA (Figure 7B).

## 4 DISCUSSION

Periodontal infection, and the key etiological periodontal pathogen, *P. gingivalis,* are considered to be associated with CVD. Through gingival ulceration during periodontitis, the bacteria and its products can spread from the subgingival pockets into the blood circulation and trigger inflammatory reactions. By isolating LDL/VLDL and HDL particles from *P. gingivalis*‐stimulated human whole blood ex vivo and using a proteomic approach, we show that proteolytic enzymes from *P. gingivalis* markedly modulate apolipoproteins.

By optical visualization, additional protein spots were found in 2D gels of the lipoproteins isolated from *P. gingivalis*‐treated blood. These are probably protein fragments produced by gingipain activity, and possibly also proteins derived from the bacteria. This study shows, for the first time, that *P. gingivalis* degrades apoE. Two apoE fragments were found and identified in isolated LDL/VLDL by mass‐spectrometry analysis. The results are in agreement with findings of the proteolytic activity on apoE by the Arg‐specific gingipains, with cleavage at Arg^33^. This generates fragments with a molecular mass of 32 kDa, a p*I* of 5.9 and 6, and a more basic position compared with the location of the original apoE isoforms seen in the 2DE gel (Figure 2, white box). Three common apoE isoforms are found in humans and the results strongly indicate that both fragments originate from the apoE‐3 isoform. ApoE‐3 is the most common isoform (allele frequency about 80% of the human population), containing a cysteine at amino acid position 130 (including the signal sequence), and an arginine at position 177. In the mass‐spectrometry data we identified peaks containing Arg^177^ in both apoE fragments, and peaks containing Cys^130^ in one of the fragments. Additionally, fragments were found in individuals missing the apoE‐4 isoform (which also contains Arg^177^). Fragments originating from apoE‐2 could be excluded because apoE‐2 differs from apoE‐3 by a single amino acid substitution and instead contains a cysteine at position 177.^30^ Optimal expression of apoE‐3 is crucial for normal lipoprotein metabolism.^30^ ApoE circulates in blood associated with VLDL, chylomicrons and HDL, and is a key regulator of plasma lipid levels by promoting the clearance of triglyceride‐rich lipoproteins by binding to the LDL receptor on hepatocytes.^30^ Another anti‐atherogenic property is that apoE mediates and facilitates cellular cholesterol efflux.^31^, ^32^ To induce LDL receptor binding, a sufficient amount of apoE in VLDL particles is needed, and the apoE molecules must be in the receptor‐active conformation with the N‐terminal helix bundle open. We speculate that the C‐terminal fragmentation induced by *P. gingivalis* induces a change in the conformation of the apoE molecule affecting its receptor interaction. This eventually inhibits the uptake of triglycerides into the lipoprotein particle and/or the liver, thereby contributing to accumulation of lipoproteins in the circulation. Indeed, the levels of LDL, triglyceride and total cholesterol are elevated in patients with periodontitis.^33^ *Porphyromonas gingivalis*‐induced fragmentation of apoE has, as far we know, not been previously reported and may be an additional mechanism involved in the modification and subsequent accumulation of lipoproteins in atherosclerotic vessels; however, this needs further investigations.

The anti‐atherogenic properties of apoE are utilized in apoE knockout mice where the atherogenic process is speeded up, enabling studies of mechanisms involved in atherosclerosis and disease development. Mice and humans lacking apoE exhibit increased levels of plasma cholesterol promoting atherosclerosis,^30^, ^34^ which confirms the importance of apoE in the metabolism of lipoproteins. Thus, our findings that *P. gingivalis* on its own modifies apoE, possibly inducing atherogenesis, has to be taken in consideration when using apoE knockout animals.

In addition, the *P. gingivalis*‐induced proteolysis of the 34 kDa full‐length apoE at Arg^33^ will produce an additional N‐terminal fragment of 2 kDa (spanning Lys^19^ to Arg^33^). No similar fragmentation products have been reported in the literature, and the identity of the key proteolytic enzyme(s) generating apoE fragments in vivo is unknown. ApoE fragmentation has been extensively studied in relation to Alzheimer's disease; therefore, it could be worth mentioning that *P. gingivalis* has been shown to be able to invade the brain,^35^ and possibly the gingipain‐induced proteolysis of lipoproteins may be a mechanism involved in the local atherogenic process and in the degenerating brain as well. Furthermore, several studies have reported a link between periodontitis and Alzheimer's disease.^36^

In agreement with our previous study, also performed in an ex vivo human blood model,^23^ we confirm that *P. gingivalis* degrades apoB‐100 on LDL/VLDL into 2 N‐terminal fragments. By using the *P. gingivalis* gingipain mutant strains, K1A and E8, we now show that the fragmentation of apoB‐100 is dependent on the arginine‐specific cysteine protease (Rgp) as no fragmentation was seen by the Rgp‐deficient mutant, E8, a strain only expressing the lysine‐specific cysteine protease (Kgp). Considering the data from the mass‐spectrometry analyses, the molecular mass values and isoelectric points of the fragments (15 kDa/7.9 and 20 kDa/7.2) correlate to the activity of Rgp gingipain with cleavage at positions Arg^158^ and Arg^207^, respectively, in apoB‐100. This also correlates to our previous study showing that *P. gingivalis‐*induced degradation of apoB‐100 was antagonized by leupeptin, a specific Rgp gingipain inhibitor.^23^ *Porphyromonas gingivalis‐*induced apoB‐100 fragmentation has also been demonstrated using western blotting techniques by exposing commercial LDL to *P. gingivalis*.^37^ Furthermore, Hashimoto et al^38^ demonstrated, in a mouse model, that *P. gingivalis* modified LDL through the Rgp‐gingipain‐mediated proteolysis of apoB‐100, which resulted in an increased uptake of LDL by macrophages and subsequent foam cell formation. The proteolysis of apoB‐100 by Rgp gingipain probably alters the surface charge of the LDL/VLDL particles generating aggregates. In correlation, Miyakawa et al,^37^ showed that the proteolytic activity of *P. gingivalis* produces aggregated forms of LDL leading to foam cell formation of macrophages, which is a key process in the formation of an atherosclerotic plaque. Dissociation of the apoB fragments from the surface of the lipoprotein particles is required to trigger aggregation of LDL,^39^ and probably this occurs after proteolysis induced by *P. gingivalis*. In addition, apoB‐100 is important for the interaction with the LDL receptor and for removing LDL and VLDL from the circulation. Interestingly, the LDL receptor (which binds apoB and apoE) in the liver does not recognize apoB‐modified LDL (eg, produced by gingipains of *P. gingivalis*), thereby resulting in the accumulation of LDL/VLDL in the circulation.^38^

Together with modified LDL, infection of macrophages with *P. gingivalis* facilitates the formation of foam cells, and the fimbriae of *P. gingivalis* are required to adhere and enter the macrophages.^40^ However, in this study, deficiency of fimbriae did not affect the proteolysis of apoB or apoE because fragments were visualized in 2DE of LDL/VLDL from whole blood treated with *P. gingivalis* mutants deficient in major (DPG3) or minor (KRX178) fimbriae (J. Lönn, S. Ljunggren, T. Bengtsson and H. Karlsson, unpublished observations).

The lysine‐specific gingipain, Kgp, of *P. gingivalis* decreased the expression of one SAA_4_ isoform (and showed a trend of reduced expression for one additional SAA_4_ isoform, *P* = .06). SAA_4_ is constitutively expressed and normally produced at a low level, and is not influenced by the acute phase response as are the other 3 SAA subtypes. The role of SAA_4_ is not well understood in human disease; however, all subtypes are associated in vivo with cholesterol control in tissues and plasma,^41^ supporting the role of *P. gingivalis* in influencing atherogenesis. However, in an in vitro situation with an enclosed system, some random fragmentations may occur that would not occur in vivo. In this study we did not find any effects of *P. gingivalis* on apoM, which was observed in our previous study.^23^ This may be explained by interindividual differences in the expression of apoM after stimulation, which we also found in this study.

W50 and its Rgp‐deficient mutant, E8, produced an additional protein spot (4685, Figure 4) on 2DE of HDL; however, the amount of protein (or number of peptides) was too low to be identified by the mass‐spectrometry analysis. This observation inversely correlated to a pattern of decreased protein expression of some proteins of HDL (including apoA‐4) identified by map‐matching, indicating that the lysine‐specific gingipain of *P. gingivalis* is involved in the degradation of proteins and their expression in HDL particles.

Different *P. gingivalis*‐induced mechanisms in blood may work synergistically to modulate the expression of lipoproteins. Previously, we have found that *P. gingivalis*‐triggered ROS production in whole blood caused oxidation of LDL proteins.^23^ Lipid peroxidation can be induced through at least 3 different mechanisms – via free radicals, via enzymatic oxidation or via free radical‐independent and nonenzymatic oxidation^22^ – although lipid peroxidation is foremost caused by free radicals from immune cells (eg, neutrophils). In this study, we show that proteolytic activity is involved in *P. gingivalis*‐induced ROS production in whole blood as preincubation with the protease inhibitor leupeptin decreased the ROS production by over 50%, and also inhibited cell aggregate formation by 75%. The production and release of superoxide and hydrogen peroxide is a powerful tool to cope with intruding pathogens. ROS is not only harmful for intruding pathogens but also causes oxidative stress in the host, recognized as an imbalance between oxidants and antioxidants. Antioxidants (eg, vitamins A, C and E, urate and bilirubin), have the ability to protect and restore ROS‐induced cell damage.^42^ Sheikhi et al^43^ have shown that the oral pathogen *Fusobacterium nucleatum* induces ROS production in neutrophils, with subsequent lipid peroxidation, which could be inhibited by vitamin E in plasma. We show similarly that *P. gingivalis* challenge of whole blood caused a reduction in antioxidant levels over time, suggesting that antioxidants are utilized to decrease cellular damage. In accordance, we show that *P. gingivalis* increased lipid peroxidation over time simultaneously with the decrease in antioxidant levels. In contrast, the gingipain mutants failed to augment lipid peroxidation; instead a decrease was seen 60 minutes after stimulation. These results correlate with the increased methionine oxidation observed in apoA‐1 in HDL of blood treated with wild‐type strains of *P. gingivalis*, while no enhanced response was induced by the mutant strains. The results indicate that both Rgp and Kgp are required to induce oxidation.

HDL prevents development of atherosclerosis by counteracting the oxidation of LDL.^44^ PON1 contributes to this atheroprotective function by neutralizing free oxygen radicals and inhibiting lipid peroxidation.^21^ However, *P. gingivalis* did not affect the activity of PON1. We have previously seen that *P. gingivalis‐*induced ROS production in whole blood reaches a maximum within 20 minutes of stimulation.^24^ Significant reducing effects on total antioxidant levels were not seen until 60 minutes after stimulation of whole blood with *P. gingivalis*. Possibly the duration of bacterial stimulation (30 minutes) was too short to generate a visible effect on PON1 activity. In addition, PON1 is synthesized in the liver, which suggests that the levels of PON1 would not change in in vitro experiments with an enclosed volume of blood.^21^

Our findings suggest that periodontal bacteria, such as *P. gingivalis*, during translocation in circulating blood, modify vascular LDL/VLDL and HDL to an atherogenic form, which supports a role of modified lipoproteins as a key link between periodontal disease and the development of atherosclerosis. Our results are based on an enclosed in vitro system, in which the involvement of new apolipoprotein synthesis in liver and the complex systemic lipoprotein metabolism in vivo are missing, as are other effects induced by inflammatory mediators. However, the whole‐blood system reflects the physiological environment in vivo, enabling interaction of the bacteria with different immune cells and plasma constituents, thus reducing confounding factors associated with isolated systems. Several studies have found periodontal bacteria, including *P. gingivalis*, in atherosclerotic plaques,^15^, ^45^ and the detection rate is several times higher in severe periodontitis compared with moderate forms.^46^ In future perspectives, it would be important to analyse the quality of the lipoproteins in clinical samples, in patients with periodontitis and with CVD, to study the situation in vivo and further investigate the mechanisms relating to the diseases.

In conclusion, our findings support a key role for the periodontal pathogen *P. gingivalis* in the link between periodontal disease and CVD, through the potent capacity of the bacterium to modify apolipoproteins and increase oxidative stress.
