# Supplementary material for: Lipoprotein modifications by gingipains of Porphyromonas gingivalis
Source: J Periodontal Res. 2018 Jan 17;53(3):403–13. doi: 10.1111/jre.12527 (PMC5969291; doi:10.1111/jre.12527)
Supplement: Supplementary file 1 [file JRE-53-403-s001.docx]

**Supporting material, Table 1.** Identified apoB-100 and apoE fragments in LDL after incubation of human whole blood with *Porphyromonas gingivalis* (5 × 10^7^ CFU/ml).

| Number | Protein | p*I* | Mass (kDa) | Masses matched | Modification | Amino-acid position |
| --- | --- | --- | --- | --- | --- | --- |
| 7107 | ApoB-100 (fragment) | 7.2 | 20 | 1012.6524 |  | 220-229 |
|  |  |  |  | 1139.5368 |  | 101–110 |
|  |  |  |  | 1212.5200 |  | 118–128 |
|  |  |  |  | 1228.5093 | Met-ox | 118-128 |
|  |  |  |  | 1666.8895 |  | 159–174 |
| 7018 | ApoB-100 (fragment) | 7.9 | 15 | 1139.5368 |  | 101–110 |
|  |  |  |  | 1212.5438 |  | 118-128 |
|  |  |  |  | 1228.5245 | Met-ox | 118-128 |
|  |  |  |  | 2153.9443 |  | 52–71 |
|  |  |  |  | 2282.0994 |  | 51–71 |
| 4209 | ApoE (fragment) | 5.9 | 32 | 1247.5763 |  | 34-43 |
|  |  |  |  | 844.4675 |  | 44-50 |
|  |  |  |  | 899.4383 |  | 51-56 |
|  |  |  |  | 1730.8585 |  | 94-108 |
|  |  |  |  | 886.4708 |  | 114-121 |
|  |  |  |  | 678.3945 |  | 133-137 |
|  |  |  |  | 1647.8127 |  | 138-152 |
|  |  |  |  | 1663.8031 | Ox: 6 | 138-152 |
|  |  |  |  | 696.4114 |  | 155-160 |
|  |  |  |  | 948.5244 |  | 177-185 |
|  |  |  |  | 616.3688 |  | 191-196 |
|  |  |  |  | 968.5482 |  | 199-207 |
|  |  |  |  | 1497.8055 |  | 210-224 |
|  |  |  |  | 817.3937 |  | 225-231 |
|  |  |  |  | 830.4277 |  | 252-258 |
|  |  |  |  | 1313.7158 |  | 259-269 |
|  |  |  |  | 1114.5805 |  | 261-269 |
|  |  |  |  | 1033.5382 |  | 270-278 |
|  |  |  |  | 1536.7292 |  | 281-292 |
|  |  |  |  | 1552.7193 | Ox: 10 | 281-292 |
|  |  |  |  | 930.4984 |  | 293-300 |
|  |  |  |  | 1620.8029 |  | 301-317 |
| 5109 | ApoE (fragment) | 6.0 | 32 | 1247.5848 |  | 34-43 |
|  |  |  |  | 844.4733 |  | 44-50 |
|  |  |  |  | 899.4429 |  | 51-56 |
|  |  |  |  | 938.4276 |  | 80-87 |
|  |  |  |  | 1730.8610 |  | 94-108 |
|  |  |  |  | 886.4774 |  | 114-121 |
|  |  |  |  | 1222.5384 | Cmet: 9 | 122-132 |
|  |  |  |  | 678.4014 |  | 133-137 |
|  |  |  |  | 1647.8139 |  | 138-152 |
|  |  |  |  | 1663.8011 | Ox: 6 | 138-152 |
|  |  |  |  | 696.4306 |  | 155-160 |
|  |  |  |  | 948.5293 |  | 177-185 |
|  |  |  |  | 968.5535 |  | 199-207 |
|  |  |  |  | 1497.8136 |  | 210-224 |
|  |  |  |  | 817.3988 |  | 225-231 |
|  |  |  |  | 839.3473 |  | 236-242 |
|  |  |  |  | 874.4536 |  | 245-251 |
|  |  |  |  | 830.4358 |  | 252-258 |
|  |  |  |  | 1313.7295 |  | 259-269 |
|  |  |  |  | 1114.5928 |  | 261-269 |
|  |  |  |  | 1033.5450 |  | 270-278 |
|  |  |  |  | 1536.7211 |  | 281-292 |
|  |  |  |  | 930.5025 |  | 293-300 |
|  |  |  |  | 1620.8061 |  | 301-317 |

**Supporting material, Table 2.**

Sequence coverage of apoE fragments identified by MS-analysis. Fragment 4209 is marked in red, and fragment 5109 also includes blue. Bold letters indicate the signal sequence:

10 20 30 40 50
**MKVLWAALLV TFLAGCQA**KV EQAVETEPEP ELRQQTEWQS GQRWELALGR
 60 70 80 90 100
FWDYLRWVQT LSEQVQEELL SSQVTQELRA LMDETMKELK AYKSELEEQL
 110 120 130 140 150
TPVAEETRAR LSKELQAAQA RLGADMEDVC GRLVQYRGEV QAMLGQSTEE
 160 170 180 190 200
LRVRLASHLR KLRKRLLRDA DDLQKRLAVY QAGAREGAER GLSAIRERLG
 210 220 230 240 250
PLVEQGRVRA ATVGSLAGQP LQERAQAWGE RLRARMEEMG SRTRDRLDEV
 260 270 280 290 300
KEQVAEVRAK LEEQAQQIRL QAEAFQARLK SWFEPLVEDM QRQWAGLVEK
 310
VQAAVGTSAA PVPSDNH

[**APOE_HUMAN**](http://www.uniprot.org/uniprot/P02649)**(P02649)**

**Whole protein:** Apolipoprotein E precursor (ApoE), Homo sapiens (Human).

**The parameters have been computed for the following feature:**

FT CHAIN 19 317 Apolipoprotein E.

**Molecular weight (Da):** 34236.68 (average mass), 34215.71 (monoisotopic mass)

**Theoretical pI:** 5.52

**Fragments:** The computation has been carried out on a user selected segment from position: 34 to position 317 in this sequence of 317 residues.

**Molecular weight (Da):** 32500.77 (average mass), 32480.83 (monoisotopic mass)

**Theoretical pI:** 6.01

**Supporting material, Figure 1.**


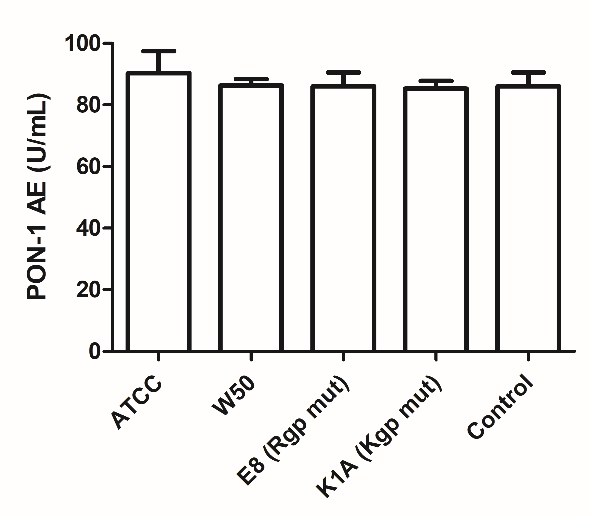


**Supplemental figure 1.** *Porphyromonas gingivalis* do not affect paraoxonase-1 arylesterase activity (PON-1 AE) in blood. Whole blood was incubated with *P. gingivalis* (5×10^7^ CFU/ml, at 37ºC, 30 min) wild type strains ATCC or W50, or the gingipain mutants E8 (lacking RgpA and RgpB) and K1A (lacking Kgp), or without bacteria (Control). PON-1 AE was measured in plasma kinetically by diluting the plasma in a salt buffer and adding a phenyl acetate solution. Produced phenol was measured and PON-1 AE was expressed in U/ml (n=3).

**Supporting material, Figure 2.**


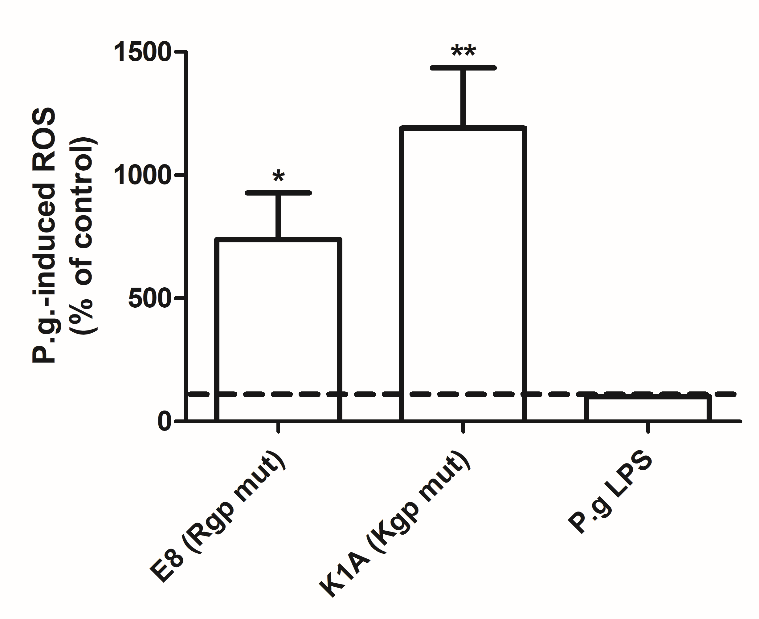


**Supplemental figure 2.** *Porphyromonas gingivalis* induced ROS-production in whole blood. Whole blood was incubated 15 min at 37ºC in the absence or presence of *P. gingivalis* (the Rgp deficient strain, E8, or the Kgp deficient strain, K1A; 1×10^7^ CFU/ml), or *P. gingivalis* LPS (1 µg/ml) for 3 hrs, under shaking. ROS production was detected by luminol-amplified chemiluminescence and presented as percentage of unstimulated-stimulated control (dotted line). Data are presented as mean ±SEM. (n=5) (**P* < 0.05, ***P* < 0.01).
